# Metabolic recoding of epigenetics in cancer

**DOI:** 10.1186/s40880-018-0302-3

**Published:** 2018-05-21

**Authors:** Yi-Ping Wang, Qun-Ying Lei

**Affiliations:** Cancer Institute, Fudan University Cancer Hospital and Cancer Metabolism Laboratory, Institutes of Biomedical Sciences, Fudan University, Shanghai, 200032 P. R. China

**Keywords:** Cancer metabolism, Epigenetics, Metabolites, Histone modification, DNA methylation, Cancer microenvironment, Nutrient availability

## Abstract

Dysregulation of metabolism allows tumor cells to generate needed building blocks as well as to modulate epigenetic marks to support cancer initiation and progression. Cancer-induced metabolic changes alter the epigenetic landscape, especially modifications on histones and DNA, thereby promoting malignant transformation, adaptation to inadequate nutrition, and metastasis. Recent advances in cancer metabolism shed light on how aberrations in metabolites and metabolic enzymes modify epigenetic programs. The metabolism-induced recoding of epigenetics in cancer depends strongly on nutrient availability for tumor cells. In this review, we focus on metabolic remodeling of epigenetics in cancer and examine potential mechanisms by which cancer cells integrate nutritional inputs into epigenetic modification.

## Background

Dysregulated metabolism is one of the most prominent features of cancer. Since the postulation of aerobic glycolysis (Warburg effect) in the early 20th century [1], metabolic reprogramming in cancer has been the subject of extensive research [2]. Cellular metabolism is reprogrammed at multiple levels in cancer: genetic, epigenetic, transcriptional, posttranscriptional, translational control, and posttranslational [3–10]. Consequently, the expression of a wide range of metabolism-related proteins, such as metabolite transporters and metabolic enzymes, are dysregulated in cancer cells [11].

Metabolism is reprogrammed in cancer cells through the action of cell-intrinsic and -extrinsic factors. Alterations in oncogenes and tumor suppressor genes cooperatively remodel metabolic pathways to satisfy biosynthetic demands of cancer cells [12]. At the same time, microenvironmental factors modulate metabolic reprogramming; these factors include nutritional [13], inflammatory, and immune elements in malignant tissue [14]. For example, metabolic activity and nutritional status of cancer cells strongly influence epigenetics, especially modifications on histone and DNA [15]. The metabolic reprogramming interacts with epigenetic regulation and signal transduction to promote cancer cell survival and proliferation [16, 17], and to influence a broad range of biological processes [18].

This review summarizes recent advances in our understanding of metabolic recoding of epigenetics in cancer, with particular emphasis on how cancer cells encode nutrient input into the epigenetic landscape.

## Main text

### Metabolites are key players in epigenetic remodeling in cancer

Cancer cells show a disordered landscape [19] of epigenetic enzymes that catalyze the addition and removal of epigenetic marks, such as modifications on histones and genomic DNA [20]. This reshaping of epigenetics is driven by alterations in epigenetic machinery as well as in the metabolic network [21].

Metabolism and epigenetics are intimately connected, as epigenetic enzymes employ various metabolic intermediates as substrates [22]. Dysregulation of metabolic pathways activates or suppresses epigenetic modifiers, leading to epigenetic remodeling. The interaction between cellular metabolism and epigenetics as well as the disease relevance of this interaction have recently been reviewed [15, 17]. The focus of the present review is how cancer metabolism modulates DNA methylation, histone methylation, and histone acetylation, as well as their connection with nutrient availability.

#### Acetyl-CoA, NAD^+^ and histone acetylation

**Fig. 1:**
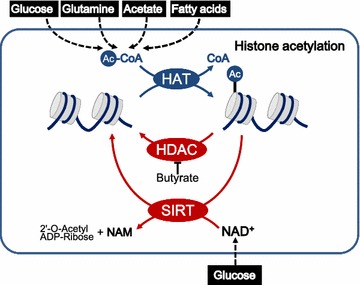
Cancer cells coordinate nutrient status with histone acetylation. Cancer cells alter histone acetylation in response to the availability of different carbon sources. *Ac-CoA* acetyl-CoA, *HAT* histone acetyltransferase, *HDAC* histone deacetylase, *SIRT* NAD^+^-dependent sirtuin family deacetylase, *NAM* nicotinamide

The most extensively investigated epigenetic marks are DNA methylation and covalent modifications of histones [23]. Histone tails are covalently modified by diverse post-translational modifications [23], of which the best understood are acetylation and methylation [20]. Histone acetyltransferases (HATs) deliver an acetyl group from acetyl-CoA to lysine residues in histones [23], whereas histone deacetylases (HDACs) catalyze the reverse reaction (Fig. 1). HDACs can be divided into two families [3]: classical HDACs directly hydrolyze acetyl-lysine [24]; SIRT-family HDACs deacetylate via an NAD^+^-dependent mechanism [25]. Histone acetylation is linked to energy metabolism since acetyl-CoA and NAD^+^ are indicators of cellular energy status (Fig. 1).

#### SAM, α-KG, oxygen and histone/DNA methylation

**Fig. 2:**
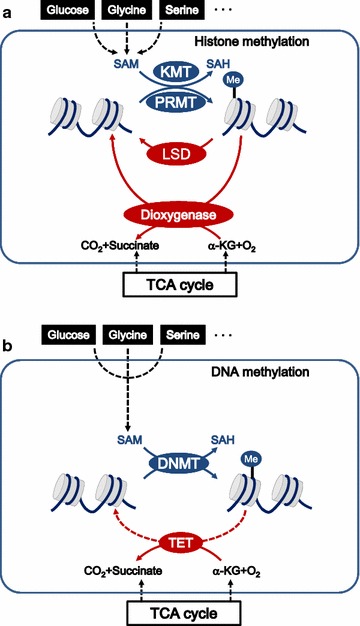
Cancer cells coordinate nutrient status with the methylation of histone and DNA. Cancer cells alter methylation of histones (**a**) and DNA (**b**) in response to nutrient status. *SAM S*-adenosyl methionine, *SAH S*-adenyl homocysteine, *KMT* lysine methyltransferase, *PRMT* protein arginine methyltransferase, *LSD* lysine-specific demethylase, *DNMT* DNA methyltransferase, *TCA* tricarboxylic acid cycle, *TET* ten-eleven translocation methylcytosine dioxygenase

Histones are methylated on lysine and arginine residues [26], and this methylation can repress or activate gene transcription [20]. Lysine methyltransferase (KMT) and arginine methyltransferase (PRMT) utilize *S*-adenosyl homocysteine (SAM) as the methyl donor in histone methylation (Fig. 2a). The reverse reaction of lysine demethylation is catalyzed by the amine oxidases lysine demethylases (LSD) 1 and 2 [27] in a reaction dependent on flavin adenine dinucleotide (FAD), as well as by an α-ketoglutarate (α-KG)-dependent dioxygenase, which produces succinate in an oxygen-dependent reaction [28] (Fig. 2a). Both α-KG and succinate are intermediates of the tricarboxylic acid (TCA) cycle, indicating a functional correlation between the TCA cycle and α-KG-dependent demethylation. The enzyme that demethylates histone arginine residues is being actively investigated [29, 30]. The protein has been proposed to be an oxygen- and α-KG-dependent dioxygenase similar to that responsible for lysine demethylation [29]. In this case, too, demethylation is linked to oxygen levels and the TCA cycle (Fig. 2a).

In humans, DNA methylation occurs predominantly at CpG islands [20]. In this process, DNA methyltransferase (DNMT) adds a methyl group—donated by SAM as in histone methylation—onto the cytosine of CpG dinucleotides (Fig. 2b). DNA methylation typically represses transcription of the marked genes, helping to stabilize the genome and promote cell differentiation [31]. The reverse reaction of DNA demethylation is catalyzed by ten-eleven translocation (TET) family enzymes, including TET1, TET2, and TET3, which are α-KG- and oxygen-dependent dioxygenases [32]. TET enzymes iteratively oxidize 5-methylcytosine (5mC) and convert α-KG into succinate (Fig. 2b).

Metabolic intermediates participate as substrates or coenzymes in nearly all epigenetic coding processes. In cancer, metabolic dysregulation interacts with nutritional status to modulate epigenetic marks on histones and DNA. This nutritional status is defined largely as the availability of carbon sources.

### Nutrient availability affects epigenetic regulation in cancer

#### Glucose availability is reflected in histone and DNA modification in cancer

Glucose and glutamine are the major carbon sources of most mammalian cells, and glucose metabolism is closely related to histone acetylation and deacetylation. Glucose availability affects the intracellular pool of acetyl-CoA, a central metabolic intermediate that is also the acetyl donor in histone acetylation [33] (Fig. 1). Glucose is converted to acetyl-CoA by the pyruvate dehydrogenase complex (PDC), which produces acetyl-CoA from glucose-derived pyruvate; and by adenosine triphosphate-citrate lyase (ACLY), which generates acetyl-CoA from glucose-derived citrate. PDC and ACLY activity depend on glucose availability, which thereby influences histone acetylation and consequently modulates gene expression and cell cycle progression [34, 35]. Dysregulation of ACLY and PDC contributes to metabolic reprogramming and promotes the development of multiple cancers, such as lung cancer [36]. At the same time, glucose metabolism maintains the NAD^+^/NADH ratio, and NAD^+^ participates in SIRT-mediated histone deacetylation [37] (Fig. 1). SIRT enzyme activity is altered in various malignancies [25, 36, 38–41], and inhibiting SIRT6, a histone deacetylase that acts on acetylated H3K9 and H3K56, promotes tumorigenesis [42, 43]. SIRT7, which deacetylates H3K18 and thereby represses transcription of target genes, is activated in cancer to stabilize cells in the transformed state [44–46]. Interestingly, nutrients appear to modulate SIRT activity. For example, long-chain fatty acids activate the deacetylase function of SIRT6, and this may affect histone acetylation [47, 48].

Glucose catabolism affects histone acetylation as well as histone and DNA methylation, since glucose-derived α-KG serves as a substrate in the reactions catalyzed by histone demethylases and TET family DNA dioxygenases [49] (Fig. 2a, b).

#### Glutamine metabolism modulates cancer epigenetics

Glutamine metabolism also contributes to the production of acetyl-CoA and α-KG, and glutamine oxidation correlates with the cell state-specific epigenetic landscape. Naive embryonic stem cells efficiently take up both glutamine and glucose to maintain a high level of α-KG to promote histone and DNA demethylation, which in turn helps maintain pluripotency [49]. Inhibition of glutamine oxidation affects histone modifications including H4K16ac and H3K4me3 in breast cancer cell lines, altering the transcription of genes involved in apoptosis and metastasis [50].

#### Acetate and other carbon sources as epigenetic metabolites

Cancer cells absorb acetate and incorporate it into histones [51]. Acetyl-CoA synthetases (ACSSs) convert acetate to acetyl-CoA, which in turn serves as a major carbon source in lower eukaryotes, but not mammals. However, glioma cells and hepatocellular cancer cells utilize acetate as an alternative carbon source to sustain acetyl-CoA production [52, 53] (Fig. 1). This compensates for the hypoxic, nutrient-poor microenvironment of solid tumors. Mammalian cells express three ACSS isozymes (ACSS1-3). The contribution of ACSS isozymes to histone acetylation varies across different cancers [54–56]. ACSS is highly expressed in glioma and hepatocellular cancer, which correlates with histone hyperacetylation [54–56]. ACLY functions as a switch and controls carbon source preference of cancer cells [57].

Other carbon sources, such as fatty acids, also regulate epigenetic modifications [58] (Fig. 1). A high-fat diet reduces the acetyl-CoA level and decreases acetylation of H3K23 in white adipose tissue but not liver. This suggests that lipids may affect cancer risk via an epigenetic mechanism, since obesity predisposes to the development of multiple cancers [59].

#### One-carbon metabolism modifies chromatin methylation

In one-carbon metabolism, the amino acids glycine and serine are converted via the folate and methionine cycles to nucleotide precursors and SAM. Multiple nutrients fuel one-carbon metabolism, including glucose, serine, glycine, and threonine [60] (Fig. 2a, b). High levels of the methyl donor SAM influence histone methylation [61], which may explain how high SAM levels prevent malignant transformation [62]. Glucose availability is encoded in methylation of H3R17 by arginine methyltransferase CARM1 [63].

#### 2-hydroxyglutarate and oncometabolites

**Fig. 3:**
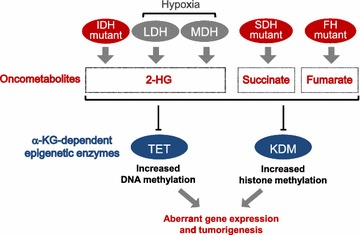
Production of oncometabolites dysregulates epigenetics in cancer. Mutations in the metabolic enzymes IDH, FH, and SDH (red) promote the generation, respectively, of the oncometabolites 2-HG, fumarate, and succinate. Hypoxia causes LDH and MDH (grey) to produce 2-HG, which acts as a competitive inhibitor of α-KG-dependent dioxygenase to deregulate DNA and histone methylation. This leads to aberrant gene expression and cancer

In cancer, genetic alteration and microenvironment perturbation modify the catalytic properties of metabolic enzymes, reshaping epigenetics. Cancer-associated mutations in isocitrate dehydrogenase (IDH) 1 and 2 confer on the enzyme the ability to produce 2-hydroxyglutarate (2-HG), which is structurally analogous to α-KG [64] (Fig. 3). 2-HG competes with α-KG to bind to the catalytic pocket of several α-KG-dependent epigenetic enzymes, suppressing their catalytic activity and leading to genome-wide hypermethylation of histones and DNA [65, 66]. The resulting aberrant gene expression promotes tumorigenesis [67, 68]. The metabolic enzymes fumarate hydratase (FH) and succinate dehydrogenase (SDH) are also frequently mutated in certain cancers [69]. Loss-of-function mutations in FH and SDH lead to accumulation of fumarate and succinate, which act as competitive inhibitors of α-KG-dependent dioxygenase [70] (Fig. 3). The oncogenic effect of α-KG, fumarate, and succinate via epigenetic regulation has led them to be named oncometabolites [15].

2-HG also accumulates in hypoxic cancer cells without IDH mutations, through a process mediated at least in part by the metabolic enzymes malate dehydrogenase (MDH) and lactate dehydrogenase (LDH). Hypoxia makes the tumor microenvironment acidic, which causes MDH and LDH to bind substrates promiscuously and generate 2-HG [71, 72] (Fig. 3). Under these conditions, more 2-HG is produced by LDH than by MDH [73]. LDH may also modulate epigenetics in cancer cells independently of 2-HG, since tumor pH is highly heterogeneous and in fact only some cancer cell lines or tumor tissues reach the pH of 6 needed to trigger promiscuous 2-HG production [74–76]. The in vivo significance of substrate promiscuity-induced 2-HG production remains to be explored.

Other metabolites show oncogenic effects in certain tissues. For example, normal colonocytes utilize butyrate as a major carbon source. Glucose is used by a subtype of colon cancer cells as the carbon source, resulting in butyrate accumulation. Butyrate further inhibits HDAC to induce histone hyperacetylation and promote the proliferation of colon cancer cells [77] (Fig. 1).

## Conclusions

**Fig. 4:**
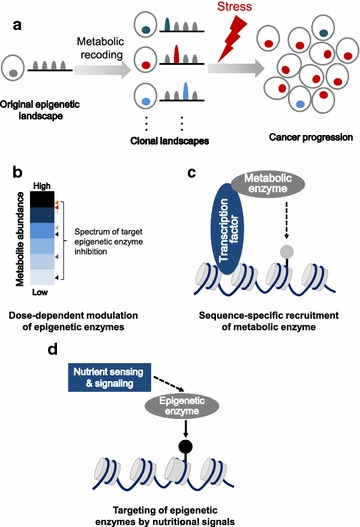
Metabolic recoding of epigenetics in cancer. **a** A model of random metabolic recoding of epigenetics in cancer. **b** Dose-dependent effect of metabolites on epigenetic enzymes. Higher accumulation of a specific metabolite affects more epigenetic targets. Half-maximal inhibitory concentrations (IC_50_) of different target epigenetic enzymes are indicated as triangles. Different colors of triangles represent different epigenetic enzymes. **c** Metabolic enzymes translocate to the nucleus, where they bind to transcription factors that carry the enzymes to specific target sequences in the genome. **d** Nutrient sensing and signaling modulate the epigenetic machinery

Cellular metabolism is highly dynamic and compartmentalized. The accumulation of certain metabolites in cancer can target epigenetic enzymes to globally alter the epigenetic landscape. Evidence suggests that this alteration can be random. For example, cancer cells containing IDH mutations show highly variable DNA hypermethylation patterns, with effects on gene transcription difficult to predict [78]. In this model of metabolic recoding of cancer epigenetics (Fig. 4a), fluctuations in the level of a metabolite produce metabolic noise and randomly modify epigenetic marks to generate diverse clonal epigenetic landscapes. This provides an opportunity for clonal selection during tumor growth, metastasis, and relapse (Fig. 4a). At the same time, recent studies have provided evidence supporting the idea that cancer-related metabolic changes lead to locus-specific recoding of epigenetic marks.

### Reign of chaos: precise epigenetic reprogramming by cancer metabolism

#### Dose-responsive modulation of cancer epigenetics by metabolites

2-HG presumably inhibits all α-KG-dependent epigenetic enzymes, but its overall effects appear to depend strongly on its intracellular concentration. Cancer cells carrying IDH mutations, for example, vary significantly in 2-HG concentration [79], and this influences the resulting epigenetic recoding. Transient expression of mutant forms of IDH suppresses the H3K9 demethylase KDM4C more strongly than other demethylases [66]. In addition, α-KG-dependent dioxygenases show diverse half-maximal inhibitory concentrations (IC_50_) of 2-HG [80]. These findings suggest that metabolic alterations in cancer cells reshape epigenetics in a manner dependent on metabolite dose (Fig. 4b).

Histones are conjugated to a large number of metabolites [81]. It is thus reasonable to expect that fluctuations of metabolites can broadly impact the epigenetic landscape. Understanding metabolism-induced epigenetic alterations requires the development of an atlas of interactions between key metabolites and epigenetic enzymes in cancer cells.

#### Sequence-specific recruitment of metabolic enzymes

Precise recoding of epigenetic marks requires recognition of a specific genomic locus or DNA sequence. Metabolic enzymes that have translocated to the nucleus may recognize specific DNA sequences by binding to transcription factors (Fig. 4c). Some metabolic enzymes translocate to the nucleus in response to stress or physiological signals. For example, glucose deprivation causes cytosolic ACSS2 to relocate to the nucleus, where it binds to transcription factor EB (TFEB). When TFEB binds to the promoter regions of lysosomal and autophagy genes, it brings ACSS2 with it; the ACSS2 produces acetyl-CoA and increases histone H3 acetylation, modulating the expression of TFEB-regulated genes [82]. In a second example, glucose starvation enhances interaction between nuclear FH and ATF2. ATF2 recruits FH to its target genes, inhibiting H3K36me2 demethylation and increasing expression of those genes, ultimately arresting cell growth [83]. Other metabolic enzymes may also translocate to the nucleus and associate with transcription factors to mediate specific epigenetic remodeling.

One hypothesis holds that the ability of nuclear ACSS2 to alter histone acetylation and of nuclear FH to alter methylation depend on high local concentrations of acetyl-CoA and fumarate, respectively, at the specific target DNA sequences [82, 83]. Testing this hypothesis requires metabolite quantification in subcellular compartments, which remains a challenging task [84]. The engineering of artificial metabolite sensors may advance locus-specific and real-time monitoring of epigenetic metabolites [85]. Studies are also needed to explore the possibility that nuclear metabolic enzymes modify epigenetic marks independently of their catalytic activity.

#### Targeting of epigenetic enzymes by nutritional signals

Nutrient sensing and signaling is a key regulator of epigenetic machinery in cancer. During glucose shortage, the energy sensor AMPK activates arginine methyltransferase CARM1 and mediates histone H3 hypermethylation (H3R17me2), leading to enhanced autophagy [63]. In addition, O-GlcNAc transferase (OGT) signals glucose availability to TET3 and inhibits TET3 by both decreasing its dioxygenase activity and promoting its nuclear export [86]. These observations strongly suggest that nutrient signaling directly targets epigenetic enzymes to control epigenetic modifications (Fig. 4d).

The nutritional status of cancer cells is highly dynamic during cancer development. How cancer cells coordinate nutrient status with epigenetic phenomena during cancer progression remains an open question.

### Concluding remarks

Our understanding of cancer metabolism has increased tremendously in the last decade. What were once considered bystander cells in the tumor microenvironment—such as cancer-associated fibroblasts [87], immune cells, and inflammatory cells [88, 89]—are now recognized as contributors to metabolic remodeling of cancer [90]. For example, oxidative cancer cells thrive on lactate in the tumor microenvironment [91], while pancreatic cancer cells depend on alanine secreted by stroma-associated pancreatic stellate cells [92]. Metabolite transport within tumor tissue and crosstalk between cancer cells and “bystander” cells cooperatively remodel cancer metabolism, suggesting an intricate and complicated regulatory network in the tumor microenvironment.

Metabolic remodeling has also been implicated in a variety of human diseases other than cancer [17, 93]. Cellular metabolism is closely related to stem cell homeostasis and differentiation [94]. Elucidating the connection between metabolism and epigenetics would provide mechanistic insights into these diseases and offer potential therapeutic opportunities for translational investigations.

## Abbreviations

HAT: histone acetyltransferase
HDAC: histone deacetylase
KMT: lysine methyltransferase
PRMT: arginine methyltransferase
SAM: *S*-adenosyl homocysteine
FAD: flavin adenine dinucleotide
α-KG: α-ketoglutarate
TCA cycle: tricarboxylic acid cycle
DNMT: DNA methyltransferase
TET: ten-eleven translocation family enzyme
5mC: 5-methylcytosine
PDC: pyruvate dehydrogenase complex
ACLY: adenosine triphosphate-citrate lyase
ACSS: acetyl-CoA synthetase
IDH: isocitrate dehydrogenase
2-HG: 2-hydroxyglutarate
FH: fumarate hydratase
SDH: succinate dehydrogenase
MDH: malate dehydrogenase
LDH: lactate dehydrogenase
TFEB: transcription factor EB
